# Interface Engineering in Organic Electronics: Energy‐Level Alignment and Charge Transport

**DOI:** 10.1002/smsc.202000015

**Published:** 2020-10-21

**Authors:** Peicheng Li, Zheng-Hong Lu

**Affiliations:** ^1^ Department of Materials Science and Engineering University of Toronto Toronto M5S 3E4 Canada; ^2^ Department of Physics Center for Optoelectronics Engineering Research Yunnan University Kunming 650091 P. R. China

**Keywords:** charge transport, electronic properties, energy-level alignment, organic interfaces, organic optoelectronics

## Abstract

Organic light‐emitting diodes (OLEDs) and organic solar cells are new members of trillion‐dollar semiconductor industry. The structure of these devices generally consists of a stack of several organic layers sandwiched between two electrodes. The electronic processes such as the energy‐level alignment at and charge transport across these interfaces play a key role to the overall performance of the organic devices. Thus, interface physics is important for design and engineering of organic devices. Herein, recent progress in energy‐level alignment at and charge transport across organic interfaces is reviewed. In addition, basic material physics of organic semiconductors such as energy levels, energy disorder, and molecular orientation is introduced. Recent progress in theories and experiments on energy‐level alignment at and charge transport across molecular heterojunctions is then discussed. Case studies of applying interface physics for guiding fabrication of ideal devices are also provided.

## Graphical abstract

The performance of organic optoelectronic devices such as organic light‐emitting diodes is dictated by the energy‐level alignment at and charge transport across the organic heterointerfaces. This review covers the modern theories and experiments on these two correlated processes. Case studies are presented to highlight their impact on the performance of organic devices.

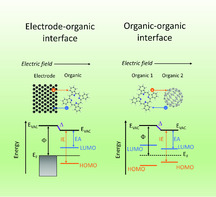

## 1 Introduction

Molecules constitute the fundamental building blocks of organic semiconductors used for making devices such as organic light‐emitting diode (OLED), organic photovoltaic (OPV) cell, and organic field‐effect transistor (OFET). OLED and OPV consist of several organic layers, sandwiched between an anode and a cathode. Efforts in the past decades have led to successful commercialization of OLED displays for televisions and smartphones. Meanwhile, a careful selection of organic compounds combined with OPV structure design has produced over 16% power conversion efficiency, which is very competitive in the commercial solar market.^[^ ^1^, ^2^, ^3^ ^]^ An OFET consists of source/drain electrode, gate electrode, gate dielectric, and organic semiconductor as the channel material. The n‐channel OFET with the field‐effect mobility exceeding 10 cm^2^ (V s)^−1^ has been achieved recently, while the p‐channel OFET with the comparable mobility is still under development.^[^ ^4^, ^5^ ^]^ The performance of these devices relies on charge carriers transporting across several organic heterointerfaces. One type of these heterointerfaces is the electrode–organic interface, where the charge carrier is injected in an OLED or OFET, or collected in an OPV cell. Another type of the heterointerfaces is the organic–organic interface, such as the interface between charge transport layer and emissive layer in an OLED, where the charge carriers are injected into the emissive layer in which excitons are formed and photons are generated.

Transport of mobile charges takes place in the frontier molecular orbitals of the organic molecules, e.g., highest occupied molecular orbital (HOMO) for holes and lowest unoccupied molecular orbital (LUMO) for electrons. Therefore, the charge transport across the organic interface is directly determined by the energy‐level alignment of the organic semiconductor at the interface. The energy‐level alignments at electrode–organic interfaces have been extensively studied in the past few decades. The HOMO binding energy with respect to the Fermi level of the electrode or the energy offset can be measured by ultra‐violet photoemission spectroscopy (UPS).^[^ ^6^, ^7^ ^]^ Other techniques such as the Kelvin probe and capacitance–voltage measurement have also been utilized to study the energy‐level alignment at organic interfaces.^[^ ^8^, ^9^, ^10^ ^]^ Several theories such as the integer charge transfer (ICT) model, induced density of states (DOS) model, electrochemical equilibrium model, and energetic disorder model have been developed to describe the energy‐level alignment at electrode–organic interface.^[^ ^7^, ^11^, ^12^, ^13^, ^14^, ^15^, ^16^ ^]^ These models are based on assumption that the electrode–organic interface topological transition is via the rather weak Van der Waals force. However, these models can be violated, albeit rare in most common interfaces in devices, if there is a strong chemical interaction at the interface.^[^ ^17^, ^18^ ^]^ Organic–organic interfaces have also been widely studied. Experimental evidences show that the theories on electrode–organic interface can also be applied to the organic–organic interface.^[^ ^7^, ^12^, ^19^, ^20^, ^21^, ^22^ ^]^

Charge transport at electrode–organic and organic–organic interfaces has drawn great attentions in the last few decades as it directly impacts the performance of the organic devices, e.g., the efficiency of the OLED. The energetic barrier can be formed at these interfaces due to the energy‐level misalignment of organic molecules. This energy barrier is generally undesirable for device applications. For instance, a large barrier at electrode–organic interface hinders the charge injection and increases the turn‐on voltage of the OLED.^[^ ^23^, ^24^ ^]^ However, when used properly, the barrier may further improve the device performance. For example, the insertion of carrier blocking layer next to the emissive layer is a common strategy to confine the excitons and thus improve the overall OLED device efficiency.^[^ ^25^, ^26^ ^]^ So the charge transport process across organic interfaces is another key aspect in constructing high‐performance organic electronic devices.

The purpose of this article is to review recent progress on energy‐level alignment at and physics of charge transport across organic interfaces. The contents of this review are as follows: first, basic materials science of organic semiconductors such as molecular orbitals, energy levels, energy disorder, and molecular orientation are briefly introduced. Second, theories and experiments of energy‐level alignments at electrode–organic and organic–organic interfaces are reviewed. Third, the theories and experiments of charge carrier injection at electrode–organic and organic–organic (including host–guest system) interfaces are discussed. Lastly, case examples of interface design and engineering for device applications are presented.

## 2 Energy Levels in Free‐Standing Molecules and Solid‐State Organic Semiconductors

**Figure 1:**
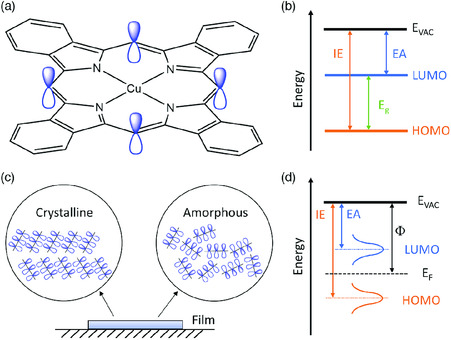
a) Molecular structure of a free‐standing *π*‐conjugated CuPc; b) schematic various energy levels of an organic molecule; c) thin film in which molecules take crystalline or amorphous packing; d) schematic various energy levels in a solid film where LUMO and HOMO are broadened.

The chemical structure of a free‐standing organic semiconductor molecule consists of alternating single and double bonds.^[^ ^27^ ^]^ The *σ*‐bonds form the molecular skeleton and *π*‐electrons are conjugated and form unpaired *π*‐electron cloud above and below the molecule plane, as shown in **Figure** **1**a. The *π*‐electronic states are delocalized within the molecule and form conduction paths for mobile charges in organic semiconductors. Similar to an atom, a molecule has its own discrete energy levels called molecular orbitals. The HOMO and LUMO are two most important energy levels for organic semiconductors because the relevant electronic and excitonic processes inside an organic device involve the HOMO and LUMO. Figure 1b shows the energy‐level diagram of a free‐standing organic molecule. Vacuum level, as shown, is defined as the energy of an unbound electron. The ionization energy (IE) of a single molecule is defined as the energy required to remove a bound electron from the molecule's HOMO to unbound state, and it can be approximated as the energy difference between the vacuum level and HOMO as per the Koopmans theorem.^[^ ^28^ ^]^ Similarly, the electron affinity (EA) of a single molecule is defined as the energy released for one electron added from unbound state into the bound state occupying molecule's LUMO, and it can be approximated as the energy difference between the vacuum level and LUMO. The energy difference between LUMO and HOMO is called energy gap (*E*_g_), which is closely associated with optical behaviors of a molecule such as the exciton formation, radiative and nonradiative decay.^[^ ^29^, ^30^ ^]^ Due to the convenience of tuning *E*_g_ by engineering the molecular structure, organic semiconductors can easily cover the whole visible spectrum, which make them extremely useful for display, lighting, and solar applications.^[^ ^31^ ^]^

An organic semiconductor film can be prepared by solution‐based (e.g., printing) or vapor‐based (e.g., thermal evaporation) methods.^[^ ^32^, ^33^, ^34^, ^35^ ^]^ The molecular packing in a solid‐state organic film can be either crystalline or amorphous, as shown in Figure 1c, depending on the deposition technique and deposition condition. For instance, single‐crystal pentacene films can be prepared by using physical vapor deposition in a horizontal glass tube under a stream of argon. Vapor‐phase deposition in vacuum can sometimes result in amorphous structure.^[^ ^36^, ^37^, ^38^ ^]^ The charge mobility of crystalline organic semiconductor is usually much higher than its amorphous counterpart due to its relatively large electronic coupling from the ordered molecular packing. However, the transport mechanism in organic crystals remains unclear. Possible charge transport regimes include polaron charge hopping, band transport, and intermediate regimes.^[^ ^39^, ^40^, ^41^ ^]^ It has been shown that significant thermal molecular motion, due to the weak Van der Waals interaction between molecules, causes the dynamic lattice disorder, which localizes the electron and prevents it from achieving band‐like transport property at room temperature.^[^ ^42^, ^43^ ^]^ For amorphous organic semiconductor with no long‐range order, the electron is heavily localized within each molecule due to the small electronic coupling, and the charge transport in amorphous organic semiconductor film can be described by carrier hopping between localized states.^[^ ^44^, ^45^ ^]^ The energy distributions of these localized states can vary from molecule to molecule, which is often called energetic disorder and is mostly caused by the molecular packing disorder inside the organic solid.^[^ ^46^, ^47^ ^]^

Energy disorder of organic semiconductor is often modeled by using a Gaussian (or Lorentz) functional DOS.^[^ ^14^ ^]^ As shown in Figure 1d, LUMO and HOMO DOS can be assumed to be a Gaussian shape. Here, the Fermi level (*E*_F_) inside the energy gap is also introduced to describe the energy required to add one electron to the organic solid film. The *E*_F_ is an elusive parameter for organic semiconductors and is dependent on the substrate on which the organic film is deposited.^[^ ^48^, ^49^ ^]^ Once the organic semiconductor is deposited, the Fermi level can be determined experimentally. The vacuum level (*E*_VAC_) for the organic semiconductor film is usually referred as local vacuum level, which is defined as the energy of a static electron just outside the film surface.^[^ ^50^ ^]^ Therefore, the vacuum level for an organic film is susceptible to its surface conditions. The energy difference between *E*_VAC_ and *E*_F_ is the work function (Φ) of the solid, which is an important parameter affecting the energy‐level alignment at organic interfaces, as will be discussed in following sections.

## 3 Energy Disorders in Thin‐Film Organic Semiconductors

**Figure 2:**
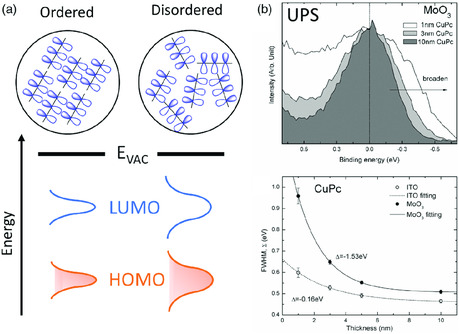
Energy disorder of organic semiconductor: a) impact of molecular packing on the energy disorder; b) experimental observed interface disorder. The top right figure is the CuPc HOMO spectra taken at various thicknesses and the bottom right figure is the HOMO width as a function of the film thickness. Reproduced with permission.^[^ ^56^ ^]^ Copyright 2019, Springer Nature.

The broadening of energy distribution or energetic disorder is characteristic of disordered organic semiconductor. The conformation variance of “soft” molecules is the major reason for the energetic disorder in organic semiconductor films.^[^ ^46^ ^]^ For amorphous organic semiconductor, each molecule inside the film may experience different packing environment, as shown in **Figure** **2**a, which leads to variant degrees of molecular deformation due to the intermolecular interaction. The deformation eventually causes a distribution in bond length, bond angle, and energy levels within each molecule and over ensemble of molecules. In addition, the variation in the dielectric environment due to the molecular packing can also induce additional variation in the energy‐level distribution, as revealed by several studies in the literature.^[^ ^51^, ^52^ ^]^ For crystalline organic semiconductor, the energy disorder is expected to be much less than its amorphous counterpart due to the well‐defined packing environment of molecules inside the solid films. However, the thermally activated molecular vibrionic motions and defects can still act as sources of energy disorder in crystalline organic semiconductors.^[^ ^42^, ^53^ ^]^

Energy disorder in bulk organic semiconductor is often assumed to be homogeneous. However, cares need to be taken for organic semiconductor films at heterointerfaces. When in contact with another material, asymmetrical interaction at the interface can cause molecule rearrangement and charge transfer, leading to additional energy disorder for organic molecules at interfaces.^[^ ^54^, ^55^, ^56^ ^]^ We have previously observed an increase in energy disorder of organic semiconductors at electrode–organic interfaces.^[^ ^56^ ^]^ As shown in Figure 2b, the HOMO of copper (II) phthalocyanine (CuPc) is broadened at the interface and its full width at half maximum (FWHM) increases with decreasing film thickness. The impact of charge transfer on energy disorder was also observed by measuring organic interfaces with indium tin oxide (ITO) and molybdenum trioxide (MoO_3_)‐coated ITO. Due to the high work function of MoO_3_ (≈6.8 eV), large number of electrons will flow from occupied HOMO states of CuPc into the electrode, creating ionized molecules near the interface. These ionized molecules will undergo structure relaxation and redistribution of energies. Meanwhile, the electric field induced by the interface dipole may further interact with the electron cloud of the molecule and induce additional structure distortion. The molecular packing is another factor because molecular packing structure can be affected by the substrate surface interaction. These factors may collectively contribute to the broadening of molecular energy levels near the interface.

## 4 Molecular Orientation and Energy Levels in Organic Semiconductors

**Figure 3:**
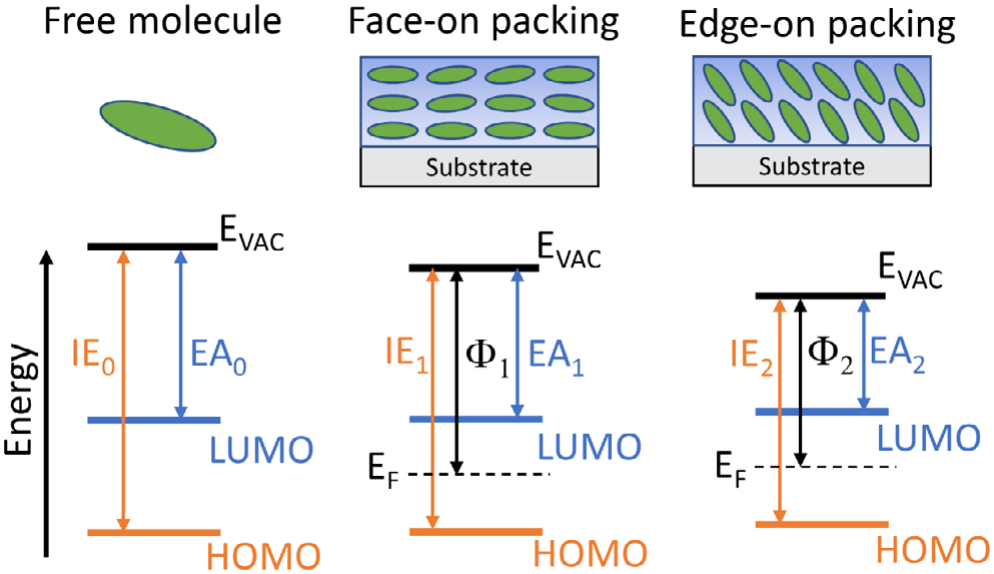
Molecular orientations in organic semiconductor. A free molecule and organic film with the face‐on or edge‐on orientation are shown. For a free molecule, the *E*_VAC_ refers to the electron energy at infinity distance. And for organic film, the *E*_VAC_ refers to the energy of a static electron just outside the film surface.

Molecular orientation is an important aspect affecting the energy levels and charge transport in amorphous organic semiconductor films. The effect of molecular orientation is usually not addressed for organic crystals because molecules at individual lattice sites can adopt various orientations. Instead, lattice anisotropy is more of interest as the properties of organic crystal heavily depend on the molecular packing along different lattice directions. For amorphous organic semiconductors, molecular orientation in a solid film is statistically disordered in most cases. However, the molecules may collectively take a preferred orientation. This is similar to molecular orientation in a liquid crystal where molecules may maintain long‐range directional order without having positional order. This preferred direction is defined as the statistically averaged molecular orientation in an amorphous organic film. Extreme orientations are shown in **Figure** **3**: face‐on orientation describes the molecule plane aligning horizontally on a substrate, and edge‐on orientation describes the molecule plane aligning vertically on a substrate. However, not all molecules may adopt the same orientation in amorphous organic thin films and it may even contain both horizontally or vertically oriented molecules. And this complex molecular alignment is often characterized by measuring the average direction of transition dipole moment of the molecule.^[^ ^57^ ^]^ The transition dipole moment is associated with the ground and excited state of the molecule and its vector has a fixed angle with respect to the molecular skeleton. Thus, the direction of transition dipole moment can be utilized to indicate the molecular alignment in the amorphous organic thin film. One of the techniques to quantify the orientation of the transition dipole moments in the organic thin film is the variable angle spectroscopy ellipsometry (VASE) which provides the information on the ratio of transition dipole moments oriented either horizontally or vertically to the substrate surface.^[^ ^57^ ^]^

*E*_VAC_ of the organic semiconductor film is dependent on the molecular orientation.^[^ ^58^, ^59^, ^60^ ^]^ As introduced before, *E*_VAC_ of the organic film is the energy of a static electron just outside the film surface, which can experience the impact of the surface dipole.^[^ ^50^ ^]^ The surface dipole is determined by the surface termination of the molecular film. The face‐on orientation leads to the *π‐*conjugated electron cloud exposed at the surface, forming a layer of surface dipoles pointing toward the substrate. This dipole layer lifts *E*_VAC_, as compared with *E*_VAC_ of a surface without the dipole layer. In contrast, the edge‐on orientation exposes the molecule's peripheral atoms on the surface. The dipole layer structure is dependent on the type of exposed atoms. For example, CuPc film with edge‐on orientation leaves the hydrogen (H) atoms exposed on the surface. It leads to the formation of surface dipoles pointing outward from the substrate due to positively charged H atoms which lost electrons to carbon C atoms. In this case, this dipole layer lowers *E*_VAC_. However, the direction of the surface dipole for edge‐on configuration can be easily switched by substituting the terminal H atoms surrounding the molecule with more electronegative atoms (e.g., copper (II) hexadecafluorophthalocyanine, F_16_CuPc).^[^ ^61^, ^62^ ^]^ Optiz et al. reported the molecular reorientation at the interface between the edge‐on CuPc and edge‐on F_16_CuPc. A face‐on CuPc‐F16CuPc layer was shown to form at the contact interface.^[^ ^63^ ^]^

**Figure 4:**
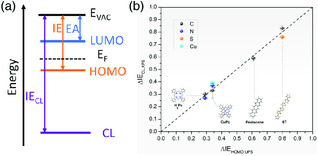
Orientation‐dependent core‐level IE: a) definition of the core‐level IE; b) experimental data comparing core‐level and HOMO IE shift due to the variance of molecular orientation. Reproduced with permission.^[^ ^83^ ^]^ Copyright 2018, the American Institute of Physics.

The variance of molecular packing leads to orientation‐dependent IE and EA for organic semiconductor films. The orientation‐dependent IE has now been recognized.^[^ ^59^, ^60^, ^64^ ^]^ The IE values obtained experimentally from traditional chemical and electrochemical methods on molecular compounds can no longer be used for solid‐state films. **Table** **1** shows the orientation‐dependent IE of several organic semiconductor films in the literature, determined both experimentally and theoretically.^[^ ^58^, ^59^, ^60^, ^64^, ^65^, ^66^, ^67^, ^68^, ^69^, ^70^, ^71^, ^72^, ^73^, ^74^, ^75^, ^76^, ^77^, ^78^, ^79^, ^80^, ^81^, ^82^ ^]^ The IE of random‐oriented molecules in films is also included for compounds such as the *N*,*N*′‐bis(naphthalen‐1‐yl)‐*N*,*N*′‐bis(phenyl)benzidine (NPB) prepared by vacuum thermal evaporation. Therefore, the IE measurement of organic semiconductor film by UPS can serve as a complementary method to evidence the molecular orientation measured by other techniques such as the VASE. The IE of core‐level electron (IE_CL_) is defined as the energy difference between *E*_VAC_ and core‐level binding energy, as shown in **Figure** **4**a. Due to the common *E*_VAC_ for valence electrons and core–shell electrons, the core‐level IE (IE_CL_) can also be utilized to measure the molecular orientation, which can be easily accessed through the X‐ray photoemission spectroscopy (XPS) measurement.^[^ ^83^ ^]^ As shown in Figure 4b, the IE_CL_ difference is identical to the IE difference (determined by UPS) between face‐on and edge‐on orientation of several organic semiconductors.

**Table 1:** Summary of IE of organic semiconductors versus molecular orientation reported in the literature. The IE measured by cyclic voltammetry is also included for comparison.^[^ ^49^, ^50^, ^51^, ^54^ ^]^

Organic material	IE_Face‐on_ [eV]		IE_Edge‐on_ [eV]		IE_Random_ [eV]	IE_CV_ [eV]
	Experiment	Theory	Experiment	Theory	Experiment	Experiment
CuPc	5.5	–	5.1	–	–	5.4
ZnPc	5.3	5.4	5.1	5.1	–	5.2
H_2_Pc	5.4	–	5.1	–	–	5.2
PEN	5.5	5.5	4.8	4.8	–	5.1
PFP	6.0	6.3	6.7	7.1	–	5.6
α‐6T	5.6	5.6	4.8	4.8	–	–
DH6T	5.3	–	4.7	–	–	5.0
F_16_CuPc	5.7	–	6.4	–	–	6.2
DIP	5.8	–	5.4	–	–	–
D5M	5.7	5.7	–	6.0	–	5.6
EL86	–	6.3	5.5	5.7	–	5.7
m‐MTDATA	–	–	–	–	5.0	4.7
CBP	–	–	–	–	6.1	5.6
NPB	–	–	–	–	5.4	5.4
C_60_	–	–	–	–	6.4	6.1

Molecular orientation affects not only the energy levels but also the charge transport characteristic inside the organic semiconductor film. For a charge carrier to migrate through the organic film perpendicularly, the face‐on molecular orientation can facilitate the charge transport because the significant *π*‐conjugated electron cloud overlapping between molecular planes makes the electron hopping along the vertical axis more easily.^[^ ^84^, ^85^, ^86^ ^]^ However, the charge hopping process can be hindered by the reduced electron cloud overlapping along the vertical direction in edge‐on or randomly oriented organic film which results in the decrease in the carrier mobility. Yokoyama et al., in 2009, conducted the time‐of‐flight measurement to determine the electron mobility of horizontally and randomly oriented oxadiazole derivatives.^[^ ^87^ ^]^ The electron mobility of the face‐on oxadiazole derivative was found to be 30 times higher than the other randomly oriented oxadiazole derivative. Therefore, the molecular orientation needs to be taken into consideration when modeling the electrical characteristic of organic optoelectronic devices.

## 5 Energy‐Level Alignment at Electrode–Organic Interface

**Figure 5:**
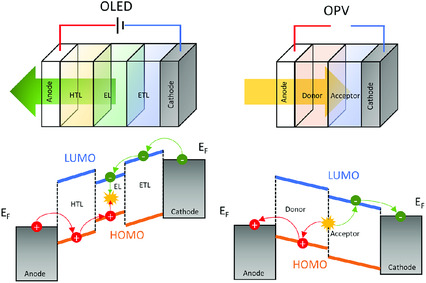
Schematic device structure of OLED and OPV, and their corresponding energy‐level diagrams.

Organic optoelectronic devices such as OLED and OPV are composed of a stack of several organic semiconductor films sandwiched between two electrodes. As shown in **Figure** **5**, an OLED typically consists of an anode, a hole transport layer (HTL), an emissive layer (EL), an electron transport layer (ETL), and a cathode. And an OPV cell consists of an anode, a photoactive layer (donor–acceptor planar or bulk heterojunction) and a cathode. The energy‐level alignment of organic semiconductor at the interfaces has significant impact on the charge injection process.

**Figure 6:**
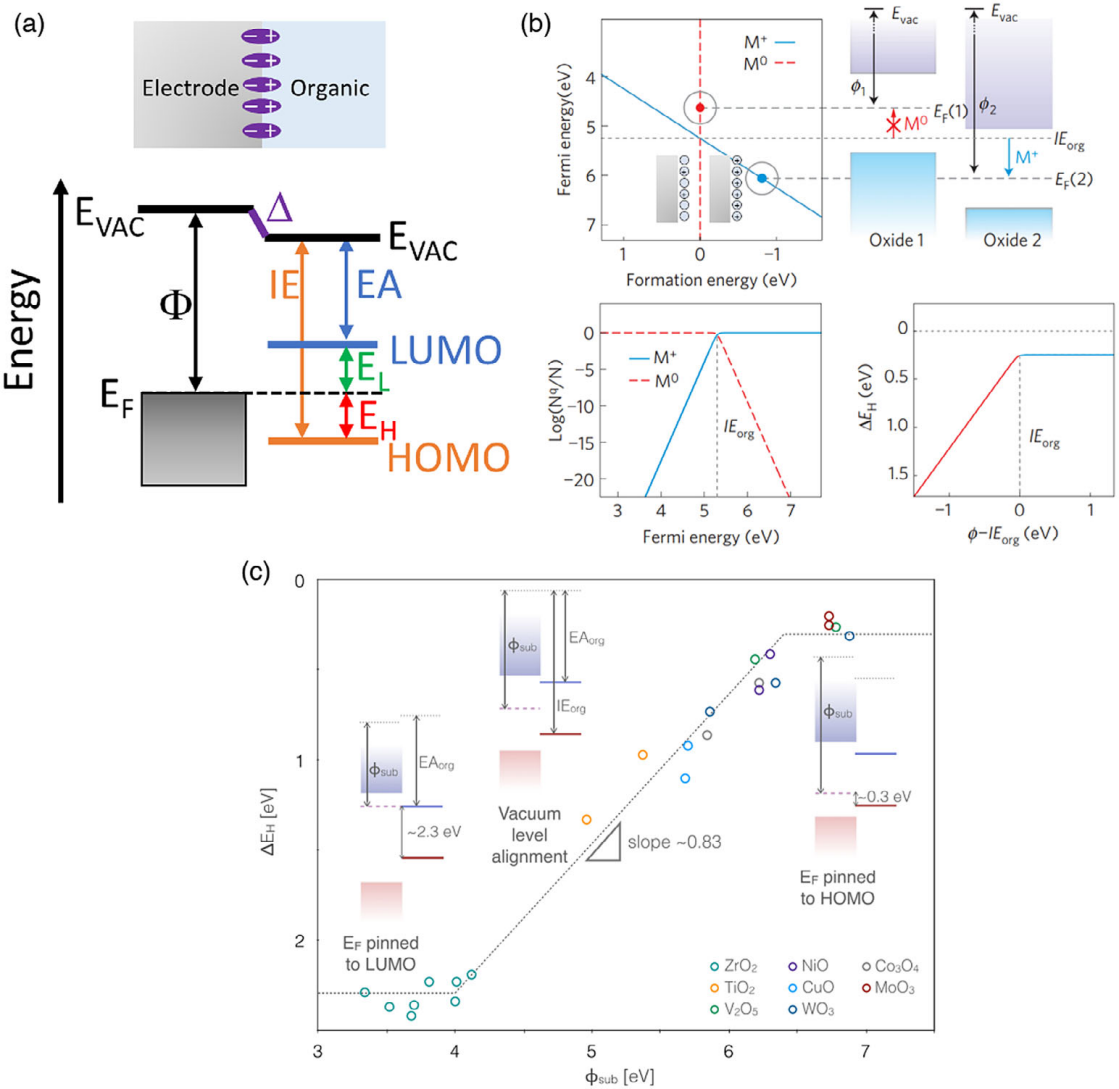
Energy‐level alignment at electrode–organic interface: a) energy‐level diagram at electrode–organic interface; b) electrochemical equilibrium model where the formation of charged molecules at the interface determines the energy‐level alignment; c) full experimental demonstration of the UELA of organic semiconductor (C_60_) on various metal oxide electrodes. Reproduced with permission.^[^ ^13^ ^]^ Copyright 2011, Springer Nature. Reproduced with permission.^[^ ^105^ ^]^ Copyright 2014, The American Physical Society.

Energy‐level alignment at electrode–organic interface has been extensively investigated in the past few decades due to its importance for the charge injection. **Figure** **6**a is a schematic electrode–organic interface energy‐level diagram. The most important parameters are the HOMO offset binding energy (*E*_H_) which is the energy difference between the electrode's Fermi level and the molecule's HOMO at the interface, and the LUMO offset (*E*_L_) defined as the energy difference between the molecule's LUMO and the Fermi level. Numerous experimental measurements of energy‐level offset have been conducted, showing that the classical Schottky–Mott rule is often not applicable to the electrode–organic interface.^[^ ^88^, ^89^, ^90^ ^]^ In Schottky–Mott limit, the *E*_H_ (or *E*_L_) is simply determined by the energy difference between the electrode work function and molecule's IE (or EA), assuming the vacuum‐level alignment at the interface.^[^ ^91^, ^92^ ^]^ However, the electron transfer across the electron–organic interface often occurs and forms an interface dipole (Δ) which shifts molecular levels, and several studies show that the Fermi level can be pinned to HOMO or LUMO no matter how the substrate work function increases.^[^ ^48^, ^89^, ^93^, ^94^ ^]^ The slope parameter, which describes how easily the *E*_L_ or *E*_H_ can be tuned by varying the substrate work function (Φ), is defined as follows^[^ ^95^ ^]^

(1) $$S=\frac{dE_{L}}{d\Phi }=-\frac{dE_{H}}{d\Phi }$$

Thus, *S* = 1 for Schottky–Mott limit and *S* = 0 for Fermi‐level pinning.

The interaction at electrode–organic interface is usually dominated by weak Van der Waals force, while the chemical bond formation, albeit rare, does exist.^[^ ^17^ ^]^ Several theories have been developed to describe the energy‐level alignment at weakly interacting electrode–organic interfaces. ICT model is one of these theories.^[^ ^7^, ^96^, ^97^, ^98^ ^]^ In this theory, two important parameters are introduced: the energy of positively and negatively charged state (*E*_ICT+_ and *E*_ICT−_) for organic semiconductors. *E*_ICT+_ is defined as the energy required to remove one electron from the molecule producing a structurally relaxed state, and *E*_ICT−_ is defined as the energy gained when one electron is added to the molecule producing a relaxed state. If the electrode work function is greater (or smaller) than *E*_ICT+_ (or *E*_ICT−_), the Fermi level will pin to the molecule's HOMO (or LUMO). If work function is between E_ICT+_ and E_ICT−_, the classic Schottky–Mott limit is achieved, i.e., the vacuum‐level alignments are aligned. It has been recently found that certain organic semiconductor follows the ICT model with an extra displacement of the vacuum level.^[^ ^99^, ^100^ ^]^ Chen et al. have studied the electrode–BPhen interface and found that the BPhen molecule follows the ICT model with an extra vacuum level shift of ≈1.4 eV. This shift was attributed to double dipole step formed at the interface.^[^ ^99^ ^]^

A 1D electrostatic model was proposed by Lange et al. to calculate the energy‐level alignment at electrode–organic interface.^[^ ^15^ ^]^ In this model, the continuous Poisson equation is utilized to link the electrostatic potential inside the film to the charge occupancy on the HOMO and LUMO of organic semiconductor

(2) $$\frac{d^{2}U}{dz^{2}}=-\frac{e}{\varepsilon_{0}\varepsilon }\{\int_{-\infty }^{+\infty }f_{H}(E,\;E_{F})g_{H}[E+eU(z)]dE\;-\int_{-\infty }^{+\infty }f_{L}(E,\;E_{F})g_{L}[E+eU(z)]dE\}$$

In Equation (2), *U* is the electrostatic potential and dependent on the distance *z* from the electrode–organic interface, *e* is the elementary charge, *ε*_0_ is the vacuum permittivity, and *ε* is the dielectric constant of the organic. The entire term inside the curly bracket refers to the net charge density at position *z*. Here, *f*_H_(*E*,*E*_F_) and *f*_L_(*E*,*E*_F_) are the Fermi–Dirac functions with the Fermi energy (*E*_F_) for hole and electron, respectively. And *g*_H_[*E* *+* *eU*(*z*)] and *g*_L_[*E* *+* *eU*(*z*)] correspond to the HOMO and LUMO DOS shifted by the electrostatic potential respectively. The HOMO and LUMO DOS are assumed to have a Gaussian shape in their study and the calculation results agree well with their experimental data. This model was further modified by considering organic molecule planes discretely. Oehzelt et al. utilized the discrete electrostatic model to further explore the impact of energy disorder (HOMO and LUMO DOS) on the energy‐level alignment at electrode–organic interfaces.^[^ ^14^ ^]^ They discovered that the degree of energy disorder in organic semiconductors is a key factor affecting the threshold value of the Φ for the Fermi‐level pinning as well as the pinning position. Khoshkhoo et al. further extended the electrostatic model by including the interface states, which successfully explained the universal Fermi‐level pinning behavior of perylene‐3,4,9,10‐tetracarboxylic dianhydride (PTCDA) on various metal substrates.^[^ ^101^ ^]^ Yang et al. have recently shown that the electrostatic model predicts well the energy‐level alignment trend of organic semiconductors, while the predicted injection barrier in the pinning region deviates from the experimental observations.^[^ ^102^ ^]^

Greiner et al. demonstrated a universal energy‐level alignment (UELA) rule at oxide–organic interface.^[^ ^15^ ^]^ The Fermi level pinned to the HOMO with an offset of ≈0.3 eV was clearly observed at interfaces where the Φ is greater than the IE of the organic semiconductor. The authors provided an analytical formulism to explain the energy‐level alignment by considering the formation of positively charged molecules at the electrode surface. The formation energy, $E^{f}(M^{q})$, of charged molecule carrying the charge *q* is given by

(3) $$E^{f}(M^{q})=E(M^{q})-E(M^{0})-qE_{F}-E_{\text{rlx}}$$

where *E*(*M* ^*q*^) and *E*(*M* ^0^) are the energy of the charged and neutral molecule, respectively. *E*_rlx_ is the relaxation energy accounting for the screening characteristics of the substrate and molecules. As shown in Figure 6b, the *E* ^f^(*M* ^*q*^) becomes negative when the electrode Φ is greater than the IE of the organic semiconductor and the formation of charged molecule becomes energetically favorable.

The equilibrium concentration, *N* ^*q*^/*N*, of charged molecules is determined by the Fermi–Dirac statistics

(4) $$\frac{N^{q}}{N}={\left\{1+g\exp[\frac{E^{f}(M^{q})}{kT}]\right\}}^{-1}$$

where *g*, *k*, and *T* are degeneracy factor, Boltzmann constant, and temperature, respectively. The *N* ^*q*^/*N* increases with the increase Φ and becomes nearly one once the Φ crosses the IE of the organic semiconductor. The Fermi level is then pinned to the HOMO because the electrostatic potential generated by charged molecules counteracts with the surface potential of the substrate. The authors also provided an analytical equation for the *E*_H_

(5) $$E_{H}=\text{IE}-\alpha \Phi +\delta \;-\frac{e^{2}\rho d}{2\varepsilon_{0}}{\sum_{i=0}^{n}\left[1+g\exp(\frac{\text{IE}-\Phi +i\beta }{kT})\right]}^{-1}$$

Here, *α* is a constant accounting for molecule's ability to screen the substrate potential. *δ* is the interface dipole caused by “push‐back” effect. *ρ* is the areal number density in the molecule plane. *d* is the distance between the substrate and the molecule plane. *ε*_0_ is the vacuum permittivity. *n* is the number of molecular planes contributing to the electrostatic potential at the interface. *β* is also a constant accounting for the surface potential decrease by individual molecule plane. Equation (5) reproduces the UELA trend by summation of *n* molecule planes contributing to electrostatic potential at the oxide–organic interface.

Ley et al. pointed out that the summation of *n* molecule layers in Equation (5), corresponding to a thick organic layer, does not agree well with the experimental condition where only about one molecule layer was deposited on the oxide. The capacitor model is therefore developed where only one molecule layer is considered.^[^ ^103^ ^]^ In this model, the oxide is treated as a perfect dielectric layer; the potential difference (Δ*φ*) across this capacitor is then given by

(6) $$\Delta \phi =\frac{eQ}{C_{\text{ox}}}$$

where *C*_ox_ is the capacitance per unit area. *Q* is the areal charge density inside the monolayer and is given by

(7) $$Q=e\rho {\left[1+g\exp\left(\frac{\text{IE}-\Phi +\Delta \phi }{kT}\right)\right]}^{-1}$$

The combination of Equation (6) and (7) solves Δϕ, and the $E_{H}$ can be simply calculated by

(8) $$E_{H}=\text{IE}-\Phi +\Delta \phi$$

However, the calculation based on one monolayer still shows a slight deviation from the UELA trend observed by Greiner et al.

Figure 6c shows a general trend of energy‐level alignment at electrode–organic interface. Three regimes can be clearly seen: 1) LUMO pinning regime due to electron transfer from the electrode to the organic, when Φ is smaller than EA; 2) elastic regime, when Φ is between EA and IE; 3) HOMO pinning regime due to electron transfer from the organic to the electrode, when Φ is larger than IE. In ideal case, the slope parameter *S* is equal to 1 in the elastic regime, indicating no electron transfer occurring at the interface. However, several experimental studies on the Schottky barrier at electrode–organic interfaces show the deviation of *S* from 1. Kahn et al. reported a *S* of 0.8 for tris(8‐hydroxyquinolinato)aluminum (Alq_3_) deposited on different metals, and Chai et al. demonstrated a *S* of ≈0.83 for fullerene (C_60_) deposited on various oxide substrates.^[^ ^104^, ^105^ ^]^ These experimental evidences show that the interface dipole does exist at electrode–organic interface even though the electron transfer is not expected given the relative position between the electrode *E*_F_ and the molecule's HOMO (or LUMO). The theory of *S* was initially developed based on the metal–inorganic semiconductor interface by Mönch in 1995, and inherited to explain the barrier formation at organic interfaces.^[^ ^11^, ^12^, ^20^, ^95^, ^106^ ^]^ At the metal–organic interface, the exponentially decayed wave function of the metal electron penetrates into the organic semiconductor and form a continuum of metal‐induced gap states (MIGS). The charge neutrality level (CNL) was also introduced inside the MIGS, of which the position is such that the integrated density of states up to the CNL accommodates the number of electrons in the isolated molecule. And the interface dipole formation depends on the alignment between metal *E*_F_ and CNL. Vázquez et al. later discovered that the CNL of organic semiconductor is insensitive to the interaction between the substrate and organic, and it can be treated as an intrinsic property for organic semiconductors.^[^ ^20^ ^]^ Therefore, the concept of CNL has also been applied to describe the electron transfer at organic–organic interface. Based on Mönch's formulism, the slope parameter at metal–organic interface is given by^[^ ^95^ ^]^

(9) $$S=\frac{A_{X}}{\left[1+(e^{2}/\varepsilon_{0}\varepsilon_{i})D_{\text{is}}\delta_{\text{is}}\right]}$$

where *A*_*X*_ = 0.86 eV/Miedema unit is the proportionality coefficient between the work function and the Miedema electronegativity of the metal, *ε*_i_ is the dielectric constant of the interface, *D*_is_ is the DOS of the MIGS around the CNL, and *δ*_is_ is the thickness of the interface dipole layer. For a “perfect” oxide layer completely suppressing the electron wave function tail of the underlying metal (*D*_is_ ≈ 0), the *S* should be around 0.86 according to Equation (9), which agrees reasonably with the observed *S* of C_60_ deposited on various metal oxide. For the organic semiconductor deposited directly on the clean metal surface, the MIGS, arising from the wave tail on the metal surface, leads to a nonzero *D*_is_ and results in the decrease in the *S*, as commonly observed in literature on metal–organic interfaces.^[^ ^104^ ^]^

White et al. have performed the reverse deposition of MoO_3_ on top of various organic semiconductors. The energy‐level alignment at these organic‐MoO_3_ interfaces was shown to obey the UELA rule, even with MoO_3_ diffusion into the organic films.^[^ ^107^ ^]^

For strongly coupled electrode–organic interface, the hybrid states between the organic and the electrode can form at the interface. Such interfaces are typically formed by depositing the organic semiconductor on top of the clean metal substrate such as copper and silver.^[^ ^107^, ^108^, ^109^, ^110^ ^]^ A chemical bond will be formed if more bonding than antibonding hybrid orbitals is occupied. The fractional charge transfer can occur when the hybrid orbitals are highly polarized. These cases involve different charge transfer characteristic from that in the ICT model, and thus the energy‐level alignment at strongly interacting interfaces usually deviates from the UELA rule mentioned earlier. While this review focuses more on the weakly interacting interface, more detailed description on the chemically reactive organic interface can be found in the recent review of Zojer et al.^[^ ^97^ ^]^

## 6 Energy‐Level Alignment at Organic–Organic Interface

**Figure 7:**
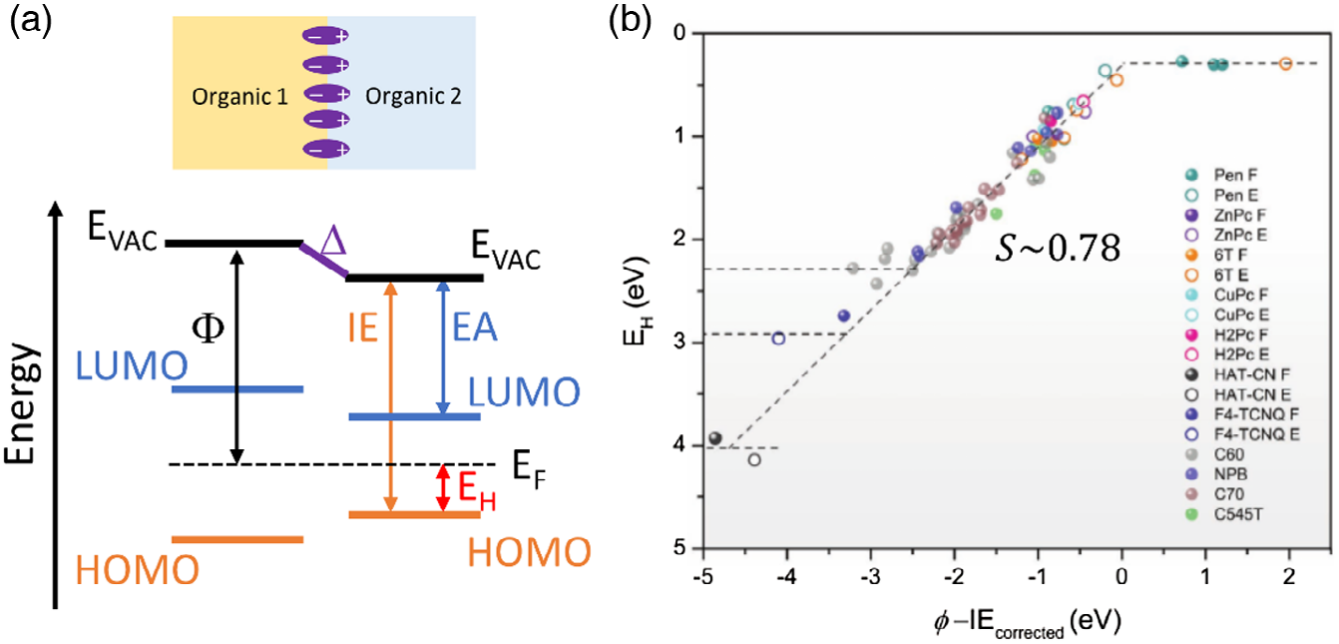
Energy‐level alignment at organic–organic interface: a) Energy‐level diagram at organic–organic interface; b) UELA at organic–organic interface. Reproduced with permission.^[^ ^19^ ^]^ Copyright 2017, Wiley‐VCH.

Organic–organic interfaces are bonded by the relatively weak Van der Waals force. This means that the theories developed based on weakly interacting electrode–organic interface can also be applied to explain the energetic behavior at organic–organic interface. According to the deposition sequence, two organic layers forming the heterojunction can be assigned as the underlying and overlying organic layer, respectively. The ICT model and electrochemical equilibrium model can be modified for the organic–organic interface by treating the underlying organic layer as a “new” substrate with an effective Φ, as shown in **Figure** **7**a.^[^ ^7^, ^19^, ^27^ ^]^ Therefore, the UELA rule should, in theory, be applicable to the organic–organic interface. The UELA trend at organic–organic interface has been experimentally studied by Li et al. who conducted UPS measurements on various organic heterojunction interfaces.^[^ ^19^ ^]^ Figure 7b shows the UELA trend at organic–organic interface, where three regimes can be clearly distinguished: 1) LUMO pinning regime due to electron transfer from the underlying organic to the overlying organic when Φ of the underlying layer is smaller than EA of the overlying layer; 2) elastic regime when Φ is between EA and IE; and 3) HOMO pinning regime due to electron transfer from the overlying organic to the underlying organic when Φ is larger than IE. Li et al. also reported empirical equations to summarize the energy‐level alignment at organic–organic interface

(10) $$E_{H}=\{\begin{array}{cc} E_{g} & \text{if}\;\Phi \leq \text{EA}\\ 0.29+S(\text{IE}-\Phi ) & \text{if EA}<\Phi <\text{IP}\\ 0.29 & \text{if}\;\Phi \geq \text{EA} \end{array}$$

where *E*_g_ is the overlying organic energy gap. As emphasized by the authors, the energy‐level alignment at organic–organic interface can be affected by the molecular orientation. This effect leads to different orientation‐dependent EAs and IEs of the same molecule. Thus, the orientation‐dependent EA and IE have to be taken into consideration when computing the energy‐level alignment at organic interfaces.

The slope parameter *S* of the elastic regime was found to be 0.78 for organic–organic interfaces, which, again, deviates from the Schottky–Mott rule.^[^ ^19^ ^]^ As compared with oxide–organic interface with a *S* of ≈0.83, the slight decrease in *S* at organic–organic interface may be attributed to a decrease in the interface dielectric constant (*ε*_i_) and an increase in the DOS of the gap states (*D*_is_) caused by the *π*‐electron interaction at the interface, as indicated by Equation (9). However, a first‐principle theory of the slope parameter at organic–organic interface has not yet been demonstrated.

The electrostatic model has also been applied to investigate the energy‐level alignment at organic–organic interface by considering different DOS of two organic semiconductors at the interface. Oehzelt et al. have utilized the discrete electrostatic model to study the energy‐level alignment at organic–organic interfaces, by assuming the energy‐level DOS of two involved organic semiconductors to be a Gaussian shape.^[^ ^22^ ^]^ In their study, the Fermi level of a supporting substrate was shown to be a key factor for simulating the energy levels at organic–organic interface because the organic heterojunction shares the electronic equilibrium with the substrate.

## 7 Charge Injection at Electrode–Organic Interface

**Figure 8:**
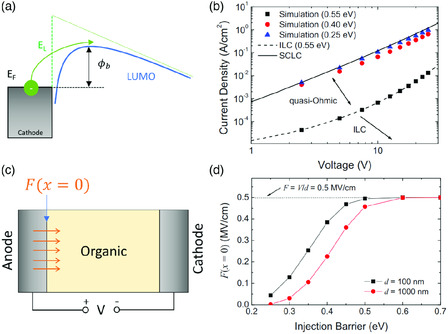
Charge injection at electrode–organic interface: a) Schematic of charge injection process at electrode–organic interface; b) calculated *J–V* characteristics of the NPB hole‐only device with various injection barrier, where the barrier of 0.25 or 0.55 eV leads to SCLC or ILC regime, and the barrier of 0.40 eV leads to quasi‐Ohmic regime; c) Schematic of a hole‐only device and the electrical field at the interface; d) field at the interface as a function of the injection barrier. Reproduced with permission.^[^ ^126^ ^]^ Copyright 2009, The American Physical Society.

Energy‐level misalignment of organic semiconductor on electrode forms the energy barrier for charge carrier injection across the interface. Thus, careful design and engineering of electrode–organic interface is key for researchers to achieve high‐performance organic devices. The process of electron (or hole) injection at electrode–organic interface is often modeled by using a combined quantum mechanical tunneling and thermodynamics of electrons (or hole) from the electrode into localized LUMO (or HOMO) states under the electric field.^[^ ^111^, ^112^ ^]^ As shown in **Figure** **8**a, the electron (or hole) must overcome the maximum injection barrier *ϕ*_b_ to be injected into the organic semiconductor. The value of *ϕ*_b_ is usually smaller than the *E*_L_ (or *E*_H_) due to the lowering of LUMO energy (or increasing of HOMO energy) by the image charge and the electric field at the interface.^[^ ^111^ ^]^

The electrode–organic contact can be categorized into Ohmic contact and Schottky contact depending on the magnitude of the *ϕ*_b_. For most organic optoelectronic devices such as OLED and OPV, the Ohmic contact is usually desired to achieve the optimal device efficiency.^[^ ^113^, ^114^, ^115^, ^116^, ^117^ ^]^ For Ohmic contact, the current flowing through an organic semiconductor is not limited by charge injection at electrode–organic interface. The current is limited by carrier mobility of the “bulk” organic semiconductor. This current is called the space‐charge limited current (SCLC) and its current density (*J*_SCLC_) is determined by the Mott–Gurney law:^[^ ^118^, ^119^ ^]^

(11) $$J_{\text{SCLC}}=\frac{9}{8}\varepsilon_{0}\varepsilon \mu \frac{V^{2}}{{d_{L}}^{3}}$$

where *V* is the applied voltage, *d*_L_ is the thickness of the organic, *ε* is the dielectric constant of the organic, and *μ* is the carrier mobility of the organic semiconductor. Many experimental measurements and theoretical calculations of *μ* in organic semiconductors show its dependence on the electric field, which is believed to be caused by the charge hopping transport between the disordered energy sites.^[^ ^120^, ^121^, ^122^, ^123^ ^]^ And this dependence on the field (*F*) is given by

(12) $$\mu =\mu_{0}\exp(\gamma \sqrt{F})$$

where $\mu_{0}$ is the zero‐field mobility and *γ* is a proportionality constant.

The Schottky contact between the electrode and organic semiconductor is formed when *ϕ*_b_ is very large and restricts the charge injection at the interface. When the electrical current in an organic semiconductor is limited by the charge injection at the electrode‐organic interface instead of the bulk property of the organic semiconductor, it is called the injection‐limited current (ILC).^[^ ^55^, ^124^ ^]^ The commonly used ILC model is the Richardson–Schottky emission where the current density (*J*_ILC_) is given by^[^ ^125^, ^126^ ^]^

(13) $$J_{\text{ILC}}=4N_{0}\psi^{2}e\mu F\exp(-\frac{e\phi_{b}}{kT})\exp(f^{1/2})$$

Here, $N_{0}$ is the density of injection sites in the organic semiconductor, *f* = *e* ^3^ *F*/4*πεk* ^2^ *T* ^2^ and *ψ* = *f* ^1^ + *f* ^1/2^–*f* ^1^ (1 + 2*f* ^1/2^)^1/2^. The formation of the Schottky contact has been shown to increase the operation voltage for OLED and result in the loss of device efficiency.^[^ ^127^, ^128^ ^]^

The current following through an electrode/organic/electrode device can be limited by both the injection at the interface and the bulk property of the organic semiconductor, when the *ϕ*_b_ at the interface is relatively small and the thermally injected charge carriers at the interface cannot be dissipated by the organic semiconductor. This type of charge injection is often referred as the quasi‐Ohmic injection. Wang et al. investigated how to distinguish SCLC, quasi‐Ohmic injection, and ILC based on the injection barrier *ϕ*_b_.^[^ ^126^ ^]^ The authors conducted a time‐domain simulation based on the drift‐diffusion and Poisson equations to study the injection property with various *ϕ*_b_. As shown in Figure 8b, the simulated *J–V* characteristic curve with a *ϕ*_b_ of 0.25 eV converges with the one determined from the SCLC model. And the simulated curve with a *ϕ*_b_ of 0.55 eV converges with the ILC model. A *ϕ*_b_ of 0.40 eV, however, leads to the *J–V* characteristic falling between the scenario of SCLC and ILC, which is in the quasi‐Ohmic regime. Another important criterion for judging the transport regime at electrode–organic interface is the electric field at the contact interface, *F*(*x* = 0), as shown in Figure 8c. SCLC requires the field vanishment at the interface, e.g., *F*(*x* = 0) = 0, which provides Ohmic contact. ILC requires a uniform field distribution throughout the device and, thus, *F*(*x* = 0) = *V*/*d*. Quasi‐Ohmic injection generally refers to 0 < *F*(*x* = 0) < *V*/*d*. The calculated field at the interface, shown in Figure 8d, shows that a *ϕ*_b_ of smaller than 0.25 eV and above 0.55 eV corresponds to a *F*(*x* = 0) of ≈0 and *V*/*d* respectively, further proving that the injected current is indeed SCLC or ILC. On the contrary, *F*(*x* = 0) between ≈0 and *V*/*d* leads to the quasi‐Ohmic behavior at the contact. Therefore, the reliable analysis of the charge injection property at the electrode–organic interface requires the knowledge of not only the injection barrier but also the electric field present at the interface.

The Ohmic contact is usually desired for the organic electronic devices. Tremendous efforts have been put into the engineering of electrode–organic interface to achieve resistance‐free contact. Recently, Kotadiya et al. proposed a universal strategy to achieve Ohmic hole injection by the insertion of a high IE interlayer between the high work function electrode and the organic transport material. The high IE organic interlayer facilitates the transport material's HOMO to pin to the electrode Fermi level.^[^ ^129^ ^]^ In the community of OFET, the insertion of self‐assembled monolayer or transition metal oxide between the source/drain and organic semiconductor has been shown to improve the charge injection and extraction process.^[^ ^130^, ^131^, ^132^, ^133^, ^134^, ^135^ ^]^ Nuzzo et al. demonstrated a graphene‐passivated nickel electrode which can be used as an efficient hole injector.^[^ ^136^ ^]^

## 8 Charge Injection at Organic–Organic Interface

Under applied electric field, charges hop from one type of molecule to another type of molecule at an organic–organic heterointerface. This hopping transport is dictated by the interface energy‐level alignment. Here, the electron and hole injection barriers at the organic–organic interface are referred to as ΔLUMO and ΔHOMO, which are defined as the energy difference between LUMOs and HOMOs across the interface, respectively. The UELA rule at organic–organic interface, as mentioned in Section 5, predicts the HOMO offset energy (*E*_H_) of the overlayer organic as a function of the work function (Φ) of the underlayer organic. Based on UELA, the ΔLUMO and ΔHOMO can be deduced

(14) $$\left\{ \begin{array}{l} \Delta \text{LUMO}=(E_{g}-E_{H})-(\Phi -{\text{EA}}^{u})\\ \Delta \text{HOMO}=E_{H}-({\text{IE}}^{u}-\Phi ) \end{array}\right.$$

where EA^u^ and IE^u^ are the EA and IE of the underlying organic semiconductor, respectively.

**Figure 9:**
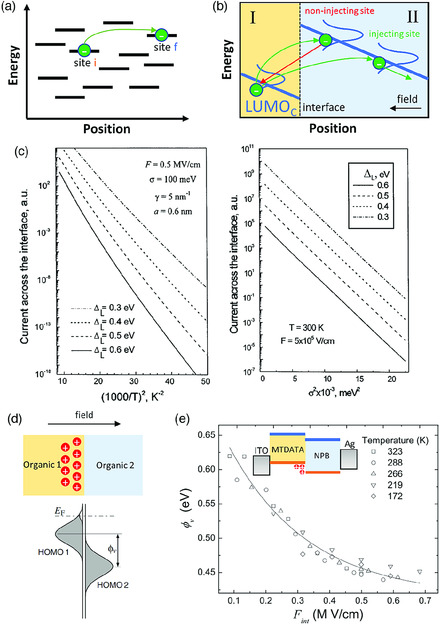
Charge injection at organic‐organic interface: a) schematic of the electron hopping between organic molecules; b) schematic of the electron injection from organic layer I into organic layer II; c) calculated injection current with various injection barrier as a function of temperature or energy disorder of the organic layer II; d) Fermi‐level realignment due to charge carriers present at organic–organic interface; e) calculated injection barrier as a function of the field at the interface. Reproduced with permission.^[^ ^137^ ^]^ Copyright 2001, American Institute of Physics. Reproduced with permission.^[^ ^139^ ^]^ Copyright 2008, The American Physical Society.

The charge injection at organic–organic interface has been commonly modeled by the hopping transport between two disordered organic semiconductors.^[^ ^137^, ^138^, ^139^ ^]^ As shown in **Figure** **9**a, the electron hopping rate is dependent on the site energy of the initial and final state, *ε*_*i*_ and *ε*_*f*_, respectively. Two commonly used formalisms to quantify the hopping rate are based on the Miller–Abrahams theory and Marcus theory. In Miller–Abrahams theory, the hopping rate (*v*_MA_) is given by^[^ ^140^ ^]^

(15) $$v_{\text{MA}}=v_{0}\exp(-2\kappa r_{if})\exp(-\frac{\varepsilon_{f}-\varepsilon_{i}+\left|\varepsilon_{f}-\varepsilon_{i}\right|}{2kT})$$

where *v*_0_ is the attempt‐to‐escape frequency, *κ* is the inverse localization radius, and *r*_*if*_ is the separation distance between initial site *i* and final site *f*. In Equation (15), the first and the second exponential term correspond to the contribution from the quantum mechanical and the thermal activation process, respectively.

The Marcus theory provides a semiclassical expression for electron transfer rate (*v*_M_) as follows^[^ ^141^, ^142^ ^]^

(16) $$v_{M}=\frac{I_{if}^{2}}{\hbar }{\left(\frac{\pi }{kT\lambda }\right)}^{1/2}\exp[-\frac{{\left(\lambda +\varepsilon_{f}-\varepsilon_{i}\right)}^{2}}{4kT\lambda }]$$

where *I*_*if*_ is the transfer integral between site *i* and *f*, *ħ* is the reduced Plank constant, and *λ* is the reorganization energy. As compared with Miller–Abrahams formulism where the molecular details are neglected, the Marcus theory introduces the *λ* to take into consideration the molecule relaxation due to the occupancy of charge carriers.

Arkhipov et al. have formulated an analytic model for charge injection at the organic–organic interface.^[^ ^137^ ^]^ As shown in Figure 9b, after an electron jumps from organic layer I to organic layer II at the interface under the field *F*, it can either jump further into II or hop back into I. Only the electrons migrating further into II contribute the injection current at the interface. For the electron to further hop through II, the currently occupied site must have the neighbor sites, which have lower site energy and thus facilitate the injection. Based on Miller–Abrahams formulism, the authors derived the average number of these injection‐facilitating neighbors, *n*(*x*, *ε*)

(17) $$n(x,\varepsilon )=2\pi \int_{0}^{x}r^{2}dr\int_{0}^{1}dz\int_{-\infty }^{\varepsilon +2\kappa kT(x-r)+eFrz}g(\varepsilon \prime )d\varepsilon \prime$$

Here, *x* is the distance of the currently occupied site from the interface, *ε* is the energy of the currently occupied site, g(*ε′*) is the DOS of organic II, and *z* = cos*θ*, with *θ* being the angle between the field and the jump direction. The probability, *w*(*x*, *ε*), of the currently occupied site having injection‐facilitating neighbors is given by the Poisson distribution

(18) w(x,ε)=1−exp[−n(x,ε)]

The injection current density, *J*_oo_, at organic–organic interface is then derived by averaging the rate of first jumps multiplied by the probability of making a second jump further into II

(19) $$J_{\text{oo}}=ev_{0}\int_{a_{i}}^{+\infty }\exp(-2\kappa x)dx\cdot \;\int_{-\infty }^{+\infty }\text{Bol}(\Delta_{L}+\varepsilon -eFx)g(\varepsilon )w(x,\varepsilon )d\varepsilon$$

In Equation (19), the function Bol(*a*) = exp[–(*a* + |*a*|)/2*kT*], *a*_i_ is the interface position and Δ_L_ is the electron injection barrier, of which the definition is slightly different from ΔLUMO as mentioned before. For convenience in theoretical simulation, the electron (or hole) injection barrier, Δ_L_ (or Δ_H_), is often defined as the energy difference of LUMO (or HOMO) DOS center between two organic semiconductors at the interface. The conversion between Δ_L_ and ΔLUMO (or, Δ_H_ and ΔHOMO) requires the knowledge of energy disorder present at the organic–organic interface.^[^ ^14^ ^]^ Assuming a Gaussian‐shaped DOS, the relationship between Δ_L_ and ΔLUMO (or, Δ_H_ and ΔHOMO) is given by

(20) $$\left\{ \begin{array}{l} \Delta_{L}=\Delta \text{LUMO}+2(\sigma_{L}^{\text{II}}-\sigma_{L}^{I})\;\\ \Delta_{H}=\Delta \text{HOMO}+2(\sigma_{H}^{\text{II}}-\sigma_{H}^{I}) \end{array}\right.$$

where *σ*_L_^I^ and *σ*_H_^I^ are the standard deviation of LUMO and HOMO DOS of organic I, and *σ*_L_^II^ and *σ*_H_^II^ are the standard deviation of LUMO and HOMO DOS of organic II. Based on Equation (19), Arkhipov et al. explored the correlation of the electron injection at organic–organic interface to the Δ_L_ and the energy disorder in organic semiconductor II. As shown in Figure 9c, even a slight increase in Δ_L_ by 0.1 eV can lead to many orders of magnitude decrease in the injection current. In addition, an increasing of energy disorder in organic II was found to hinder the charge injection. Thus, a control of injection barrier and the energy disorder is the key to engineer the charge transport at organic–organic interface.

However, the injection barrier is not always a constant and is dependent on the charge carrier concentration at both sides of organic–organic interface.^[^ ^138^, ^139^ ^]^ As pointed out by Tsang et al., this variable barrier can be explained by the dynamic Fermi‐level alignment at organic–organic interface.^[^ ^139^ ^]^ Like doping in semiconductor, the occupancy of charge carriers in organic semiconductors shifts their Fermi level within the energy gap. The variable barrier is resulted from the realignment of the Fermi level between two organic semiconductors at the interface, as shown in Figure 9c. As shown in Figure 9d, the theoretical calculation by Tsang et al. shows an increasing electric field at the interface leads to increase of carrier concentration, which results in the reduction of the hole injection barrier.

Another possible injection mechanism at organic heterojunction interface is the direct electron tunneling. When the HOMO of the organic I aligns with the LUMO of the organic II, the direct electron tunneling from the HOMO of I into the LUMO of II is possible under the electric field. A typical example of this heterojunction is the C_70_/pentacene heterojunction, which has been studied by Guo et al.^[^ ^143^ ^]^ This kind of heterojunction structure is typically used as the charge injecting layer for OLEDs and the charge generation connecting layer for tandem OLEDs.^[^ ^144^, ^145^, ^146^ ^]^

## 9 Charge Transport in Host–Guest Organic Semiconductor

**Figure 10:**
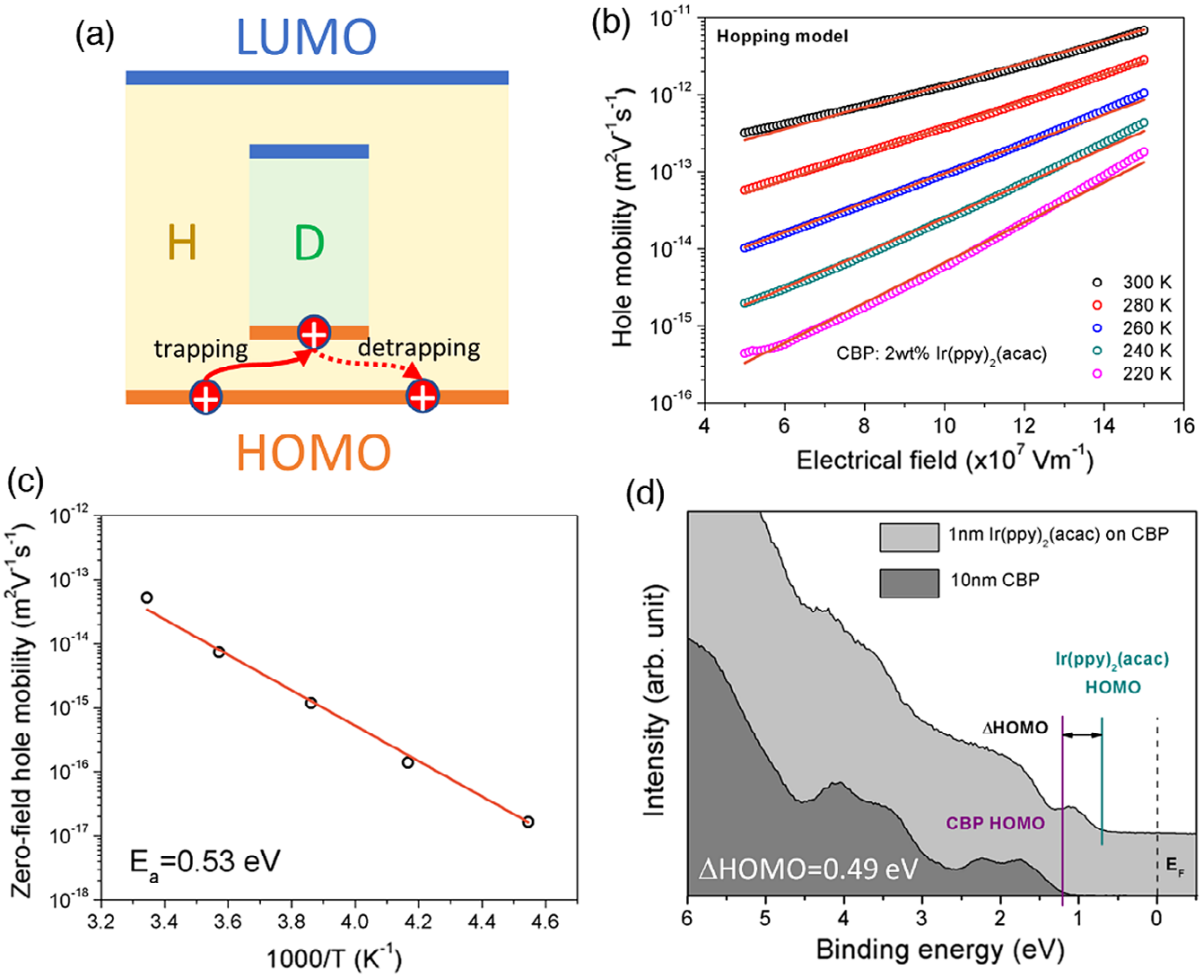
Charge transport in lightly doped host–guest semiconductor: a) schematic of hole trapping and detrapping process in host–guest semiconductor; b) experimentally measured hole mobility of host:guest organic semiconductor (CBP:2 wt% Ir(ppy)_2_(acac)) as a function of the electrical field under various temperature; c) zero‐field hole mobility as a function of the temperature; d) UPS spectra of host/guest interface (CBP/Ir(ppy)_2_(acac)). Reproduced with permission.^[^ ^151^ ^]^ Copyright 2020, Wiley‐VCH.

Host–guest organic semiconductor is a mixture of host organic matrix and guest molecule. This host–guest structure has been widely used for the emissive layer of an OLED.^[^ ^147^, ^148^ ^]^ Charge transport in a host–guest organic material is dependent on the energy‐level alignment at the host–guest interface and the guest concentration.^[^ ^149^, ^150^, ^151^ ^]^ As shown in **Figure** **10**a, the hole (or electron) can be trapped by the guest molecule if the HOMO (LUMO) energy of the guest molecule is higher (or lower) than that of the host.

The conduction mechanism of the trapped hole varies with the guest concentration. For low‐concentration system with large intermolecule distance of guests, the hopping transport between guests is unlikely to occur due to the exponential decay of hopping rate with the distance, and the conduction of the trapped hole requires overcoming the energy barrier between host and guest under applied electric field *F*.^[^ ^149^, ^150^, ^151^, ^152^ ^]^ This so‐called detrapping process is thermally activated and the hole mobility (*μ* ^H^) in this case can be expressed as follows^[^ ^151^ ^]^

(21) $$\mu^{H}=\mu_{0}^{H}\exp(-\frac{\Delta \text{HOMO}-eFd^{H}}{kT_{\text{eff}}})$$

where $\mu_{0}^{H}$ is a prefactor, ΔHOMO is the hole injection barrier (as defined in Section 7) at the host–guest interface, and *d* ^H^ is the hopping distance of the detrapping process. *T*_eff_ is the effective temperature determined by 1/*T*_eff_ = 1/*T*–1/*T*_MN_, where *T*_MN_ is the Meyer–Nedel temperature. Equation (21) neglects the interaction between the electron and its correlated positive ion trap under the assumption that the ion trap can be efficiently filled by the electron of the adjacent host under the field. The temperature‐dependent hole mobility of several host–guest organic semiconductors can be extracted and one example is shown in Figure 10b. As shown in Figure 10c,d, fitting Equation (21) to experimental data yielded the ΔHOMO of the host–guest organic semiconductors, which agrees well with the ΔHOMO directly measured by UPS.

**Figure 11:**
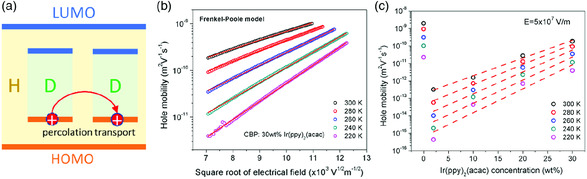
Charge transport in heavily doped host–guest semiconductor: a) schematic of hole percolation transport through guest sites; b) experimentally measured hole mobility of host:guest organic semiconductor (CBP:30 wt% Ir(ppy)_2_(acac)) as a function of the electrical field under various temperature; c) the hole mobility of host:guest organic semiconductor (CBP:Ir(ppy)_2_(acac)) at the field of 5 × 10^7^ V m^−1^ as a function of the guest concentration. Reproduced with permission.^[^ ^151^ ^]^ Copyright 2020, Wiley‐VCH.

With increasing guest concentration, the intermolecular distance of guests becomes sufficiently small, allowing the trapped hole to hop between the guest molecules, as shown in **Figure** **11**a. This enables percolation charge transport through the guest molecules. In this case scenario, the charge transport in a host–guest system can be treated as the charge migration in a “pure” guest material. The mobility of a pure organic semiconductor usually follows a Frenkel–Poole type formulism, where the mobility is exponentially dependent on the square root of the field, as shown in Equation (12).^[^ ^121^ ^]^ We have also studied the hole mobility (*μ* ^FP^) of high‐concentration host–guest system and found that the Frenkel–Poole formulism can be applied to describe the mobility, as shown in Figure 11b. The Frenkel–Poole type mobility is given by

(22) $$\mu^{\text{FP}}=\mu_{0}^{\text{FP}}\exp(-\frac{E_{a}^{\text{FP}}-\gamma \sqrt{F}}{kT_{\text{eff}}})$$

where *μ*_0_^FP^ is the prefactor and *E*_a_^FP^ is the activation energy of the Frenkel–Poole type transport. The mobility of the charge in the percolation regime is dependent on the guest concentration. With the increasing guest concentration, the electron wave function overlapping becomes more and more significant, which further increases the charge mobility, as shown in Figure 11c.

For moderate guest concentration, the detrapping process and percolation transport can occur at the same time. The commonly used method to simulate the charge transport in a general host–guest system is to consider charge carriers migrating through a lattice with the site energies drawn randomly from a bimodal Gaussian DOS, *g*_HG_(*ε*)^[^ ^149^ ^]^

(23) $$g_{\text{HG}}(\varepsilon )=(1-c)g_{H}(\varepsilon )+cg_{G}(\varepsilon )$$

where *g*_H_(*ε*) and *g*_G_(*ε*) are DOS of host and guest, respectively, and *c* is the relative concentration of the guest sites. The Pauli master equation describing the charge hopping process is used in the simulation

(24) $$\sum_{i\neq j}[-v_{ij}p_{i}(1-p_{j})+v_{ji}p_{j}(1-p_{i})]=0$$

where *p*_*i*_ (or *p*_*j*_) is the probability that site *i* (or *j*) is occupied by a charge. And *v*_*ij*_ (or *v*_*ji*_) is the hopping rate from site *i* to *j* (or, from *j* to *i*) determined by Miller–Abrahams formulism, as shown in Equation (15). Through solving *p*_*i*_ by iteration procedure, the charge mobility (*μ*) can be calculated from

(25) $$\mu =\frac{\sum_{i,j,i\neq j}v_{ij}p_{i}(1-p_{j})R_{ij}}{(\sum_{i}p_{i}/V_{0})V_{0}F}$$

where *R*_*ij*_ is the displacement between site *i* and *j* in the direction of the electric field *F*. And *V*_0_ is the volume of the lattice. The advantage of this numerical method is that it is applicable to the host–guest system of any guest concentrations. Yimer et al. have conducted the simulation based on the master equation, which shows a good agreement with our experimental findings.^[^ ^149^ ^]^

Sanderson et al. have recently implemented a kinetic Monte Carlo simulation to study the impact of dopant concentration on the transport process in a host–guest system. They found that the carrier mobility shows a minimum at the guest concentration of 10 wt%, which is attributed to the formation of connected guest clusters that create effective trap sites.^[^ ^153^ ^]^

## 10 Case Applications

Understanding the physics behind the energy‐level alignment at and charge transport across organic interfaces is very useful for engineering of organic optoelectronic devices. In this section, case examples on correlation between the interface energetics and electrical characteristic of organic devices are presented.

### 10.1 Mobility Measurement

**Figure 12:**
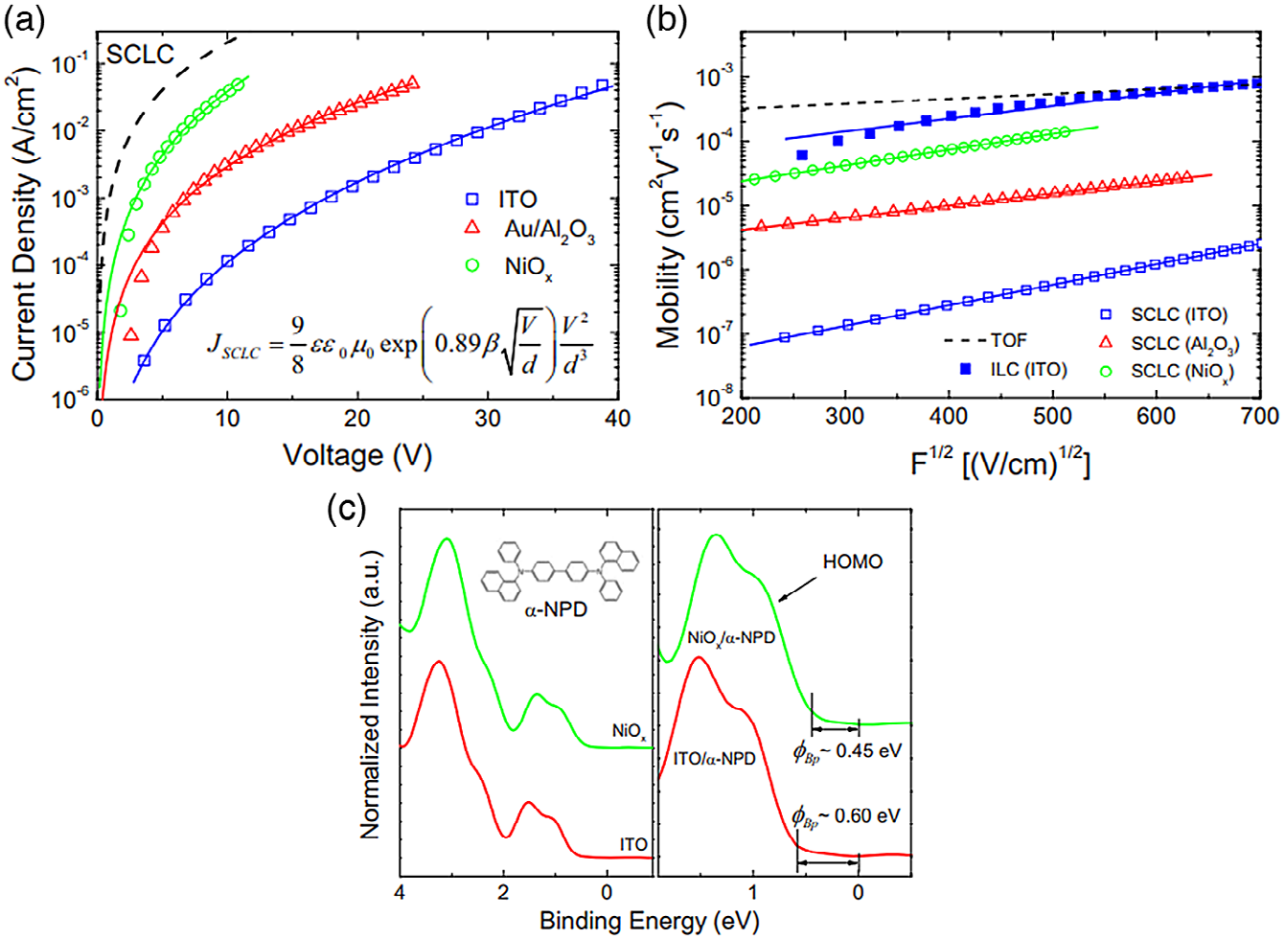
Misinterpretation of the charge mobility by using SCLC method on the device without Ohmic contact: a) experimentally measured *J–V* characteristics of NPB hole‐only devices fabricated on various electrodes; b) deduced hole mobility by using SCLC model as a function of the electrical field, showing a significant deviation from the mobility determined by ToF method; c) UPS valence band spectra of NPB deposited on various electrodes showing large injection barriers. Reproduced with permission.^[^ ^154^ ^]^ Copyright 2010, American Institute of Physics.

Charge carrier mobility is an important parameter for organic semiconductors. The mobility can be measured by techniques such as the SCLC, admittance spectroscopy (AS), time‐of‐flight (ToF), and dark‐injection transient current (DITC) method.^[^ ^154^, ^155^, ^156^, ^157^, ^158^, ^159^ ^]^ As compared with the transient method (e.g., ToF and DITC), the SCLC and AS method are more versatile and can be conducted on simple electrode/organic/electrode devices.^[^ ^155^, ^159^ ^]^ This, however, does not mean that the extracted mobility by these two methods is always reliable. The applicability of SCLC and AS method requires that the contact between the electrode and organic semiconductor has to be Ohmic. The knowledge of the injection barrier and the electric field at the interface is, therefore, key for the reliable SCLC and AS measurement of charge mobility. Wang et al. have measured *J–V* characteristics of NPB hole‐only devices fabricated on different injection electrodes.^[^ ^154^ ^]^ As shown in **Figure** **12**a, even though all *J–V* curves can be fitted by using SCLC model, they never reach the theoretical SCLC curve with the hole mobility of NPB determined by the ToF measurement. The application of SCLC model without the knowledge of interface energetics leads to underestimation of charge mobility, as shown in Figure 12b. Further UPS measurement shows that the injection barrier of 0.45 and 0.6 eV exists at the nickel oxide–NPB interface and ITO–NPB interface, as shown in Figure 12c. The former barrier leads to the quasi‐Ohmic injection and the latter leads to ILC at the electrode–organic interface.^[^ ^154^ ^]^ Helander et al. have developed high work function chlorinated ITO (Cl‐ITO) electrode, which can be used as the Ohmic hole injection electrode for reliable mobility measurement.^[^ ^159^ ^]^ The authors conducted the AS on the NPB hole‐only device based on Cl‐ITO electrode and the measured mobility shows a good agreement with the one extracted from ToF measurement. Therefore, genuine Ohmic electrode/organic interface is critical to reliable measurement of charge mobility by SCLC and AS method.

### 10.2 Engineering Electrode–Organic Interface for Efficient OLED

**Figure 13:**
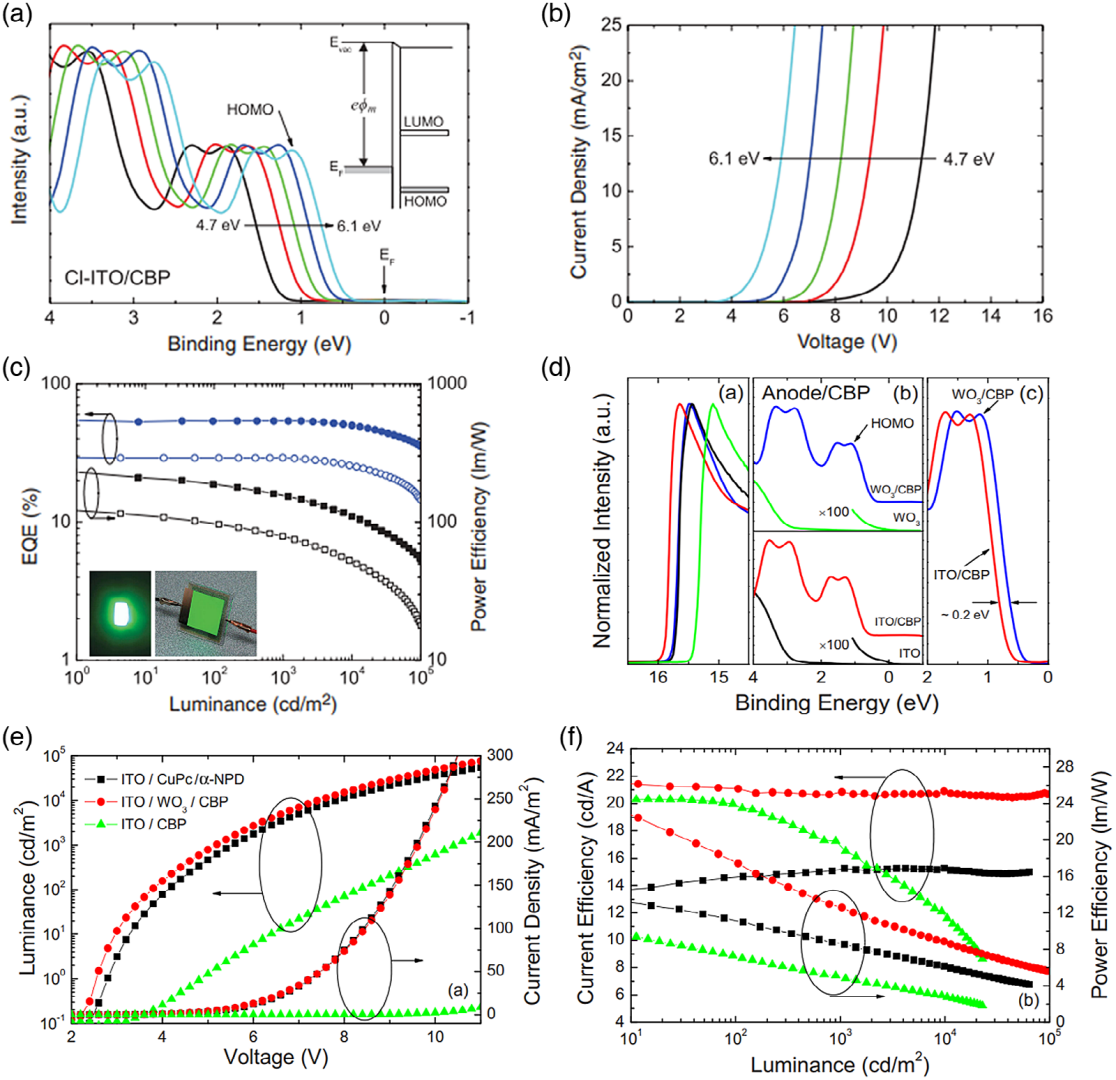
Engineering ideal electrode–organic interface for OLEDs: a) UPS spectra of CBP deposited on chlorinated ITO electrode showing a reduction of the injection barrier due to the increasing work function of ITO; b) *J–V* characteristic of OLEDs fabricated on chlorinated ITO showing the reduced operating voltage due to enhanced injection at the electrode–organic interface; c) EQE of OLED fabricated on chlorinated ITO; d) UPS spectra of CBP deposited on the bare ITO and the ITO coated with WO_3_; e) *J–V* and luminance–*V* characteristics of OLEDs fabricated on the ITO with and without the WO_3_; f) current efficiency and power efficiency as a function of the luminance for OLEDs made on the oxide‐coated ITOs. Reproduced with permission.^[^ ^160^ ^]^ Copyright 2011, American Association for the Advancement of Science. Reproduced with permission.^[^ ^165^ ^]^ Copyright 2010, American Institute of Physics.

Charge injection at electrode–organic interface is one of the key parameters impacting device performance of OLEDs. The minimization of this interface barrier facilitates the charge injection, thus reducing the operating voltage and increasing the device efficiency. The common strategy for reducing the barrier is to increase (or reduce) the work function of the anode (or cathode) by surface modification of the electrode. This can be done by chemical treatment on the electrode surface.^[^ ^160^, ^161^, ^162^ ^]^ For example, Helander et al. demonstrated that surface chlorination on ITO electrode can significantly increase the work function of ITO, from 4.7 to 6.1 eV.^[^ ^160^ ^]^ The increase in ITO work function leads to a significant reduction in the hole injection barrier at the electrode–organic interface as shown in **Figure** **13**a, and a drastic decrease in the OLED operating voltage as shown in Figure 13b. The external quantum efficiency (EQE) of the corresponding device is increased over 50%, as shown in Figure 13c. The other method to increase the anode work function is to deposit a high work function thin surface modification layer.^[^ ^24^, ^163^, ^164^ ^]^ Some transition metal oxides are often used as a surface modification layer for the anode due to their high work functions. Wang et al. showed that the deposition of the transition metal oxide such as MoO_3_ and tungsten trioxide (WO_3_) on top of the ITO can efficiently reduce the hole injection barrier, as shown in Figure 13d.^[^ ^165^ ^]^ This strategy leads to efficient hole injection at electrode–organic interface and great enhancement in the device efficiency, as shown in Figure 13e,f. For cathode/ETL interface, surface modification can also lead to the improvement in device performance. The common approach to reduce the cathode/ETL injection barrier is to deposit a low work function material, such as calcium (Ca) and ytterbium (Yb), on ETL.^[^ ^166^ ^]^ A thin layer of metal fluoride (e.g., lithium fluoride and cesium fluoride) has also been shown to improve the electron injection in OLED.^[^ ^167^, ^168^, ^169^ ^]^ Piromreum et al. attributed this improvement to the doping in organic transport layer by the metal cation released from the chemical reaction between the cathode and the fluoride.^[^ ^169^ ^]^ Kim et al. proposed that the improved performance originated from a lowering of the electron injection barrier due to the repositioning of the cathode Fermi level.^[^ ^170^ ^]^ Yuan et al. showed the anion of fluoride salt can act as a n‐type dopant which donates electrons to the organic semiconductor and improved the electron injection at the interface.^[^ ^171^ ^]^ Helander et al. argued that the formation of strong interface dipole due to the intrinsic dipole moment of the metal fluoride lowers the electron injection barrier at metal–organic interface.^[^ ^172^ ^]^

### 10.3 Charge Injection from Charge Transport Layer into Emissive Layer in OLED

**Figure 14:**
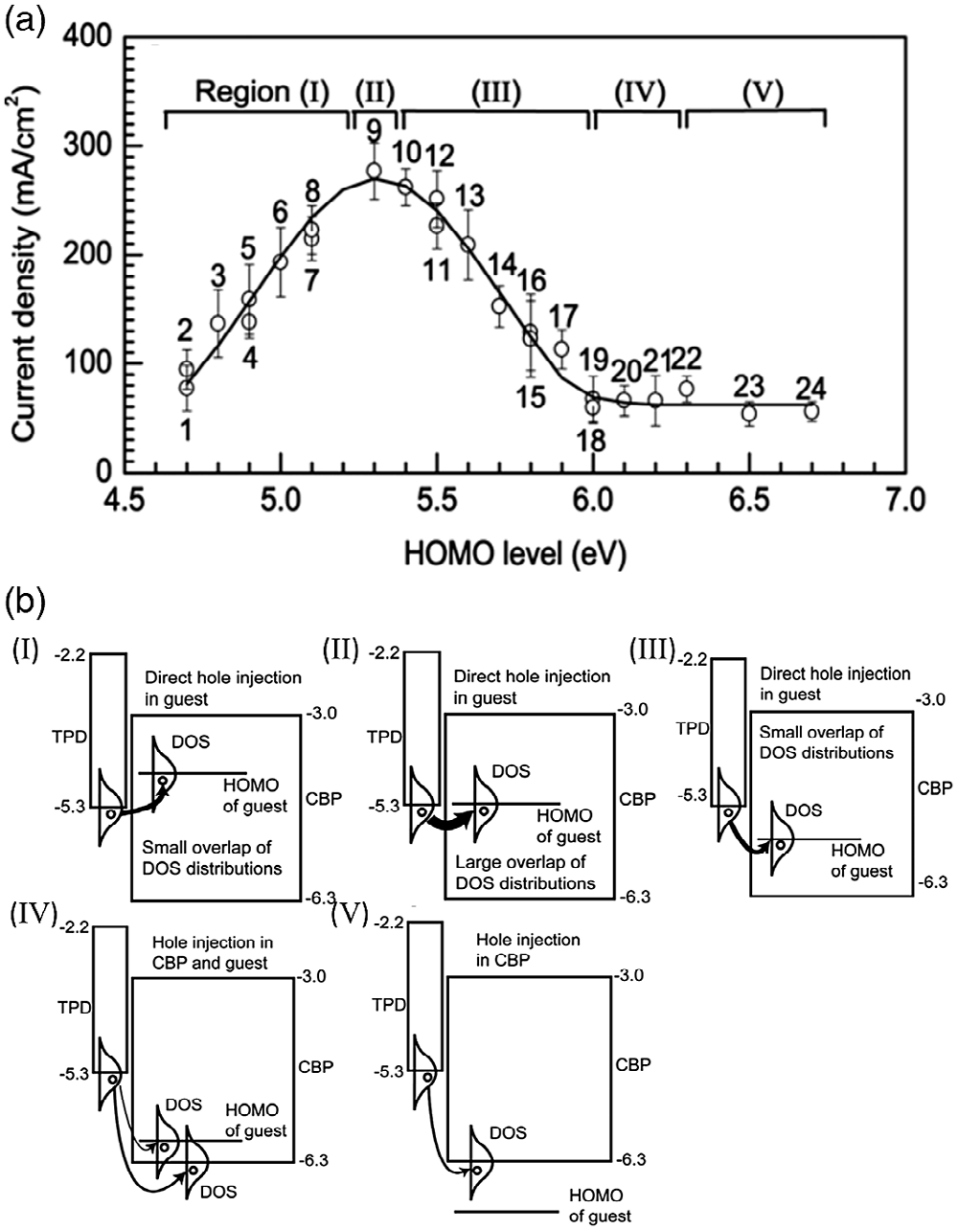
Charge injection at HTL/EL interface in OLED: a) current density (under 10 V bias) of OLEDs doped with various guest molecules as a function of the guest HOMO levels; b) schematic of charge injection process at the HTL/EL interfaces. Reproduced with permission.^[^ ^173^ ^]^ Copyright 2007, Elsevier.

In addition to the electrode–organic interface, charge transport across the organic–organic interface can also impact OLED performance. One example is the charge transporting layer (TL)/emissive layer (EL) interface. As the EL is made of host doped with guest molecules, the charge injection barriers at this interface involve alignment of TL energy level to that of host and guest. Matsushima et al. have conducted *J–V* measurements of OLEDs having various guest molecules doped in a CBP host.^[^ ^173^ ^]^ The HTL and the host material in their devices are *N*,*N′*‐bis(3‐methylphenyl)‐*N*,*N*′‐diphenylbenzidine (TPD) with a HOMO energy of ≈5.3 eV and 4,4′‐bis(*N*‐carbazolyl)‐1,1′‐biphenyl (CBP) with a HOMO energy of ≈6.3 eV. As shown in **Figure** **14**a, the current densities of these OLEDs are controlled by the hole injection at the HTL/EL interface. The current density follows a clear trend as a function of the guest HOMO energies. Figure 14b shows that, when the HOMO energy of the guest molecule is smaller than that of TPD, the direct injection from TPD to the guest molecule is energetically favorable. The current density increases, due to an increasing overlap of DOS between TPD and the guest molecule, until the HOMO of the guest reaches that of TPD. A further increase in HOMO energy of the guest leads to a decrease in DOS overlapping between the TPD and the guest and thus a reduction of charge injection. When the HOMO of the guest is greater than that of the CBP, the hole injection to the host molecule dominates, resulting in an independence of current density on the HOMO energy of the guest molecule. These experimental data clearly show the linkage between energy‐level alignment at the HTL/EL interface to OLED performance.

## 11 Conclusion

In summary, we reviewed recent progress in theories and experiments of energy levels at and charge transport across organic semiconductor interfaces. The key points are summarized as follows: 1) Energy disorder is intrinsic for organic semiconductor thin films. The additional energy disorder can occur at heterojunction interfaces. The interface energy disorder impacts charge distribution and consequently energy‐level alignment. 2) Energy levels such as ionization energies and electron affinities of molecules in solid films can vary significantly, depending on molecular packing structure, i.e., molecular orientation in films. One must take this into account in computing interface energy barrier. 3) For energy‐level alignments of organic overlayers on underlayer substrates (such as electrode, charge injection oxides, and charge transporting organics), an universal rule, came with a set of empirical equations, has been developed to predict interface energy offsets using substrate work function, the IE and EA of the overlayer organic semiconductor. As per this rule, electrons spontaneously transfer from the organic layer to the substrate (or, from the substrate to the organic layer), leading to Fermi level pinned to the HOMO (or LUMO), if the substrate work function is higher (or lower) than the IE (or EA) of the organic semiconductor. When the substrate work function is in‐between the IE and EA of the organic layer, the HOMO (or LUMO) offset binding energy scale linear elastically according to the substrate work function with a slope parameter of 0.78 and 0.83, for organic–organic and electrode–organic interface, respectively. 4) Charge transport across organic interface is dictated by the energy‐level alignment. For electrode–organic interface, the injection barrier and the interface electric field should be collectively used to judge the contact to be Ohmic, quasi‐Ohmic, or Schottky contact. 5) Charge transport in host–guest organic semiconductor involves charge trapping, detrapping, and percolation process. At very low guest concentration, thermally activated charge conduction involves trapping–detrapping between guest–host molecules. At high guest concentration, trapped charge percolating from one guest molecule to another take over the conduction. 6) Calculation of charge carrier mobility from current–voltage measurement of single carrier device is reliable only if the electrode–organic interface forms true Ohmic contact, i.e., the interface barrier is less than 0.2 eV for NPB. 7) As a case example, interface barrier engineering via high work function chlorinated ITO electrodes is shown to produce some of the very best performing OLEDs.

## Conflict of Interest

The authors declare no conflict of interest.
